# New insights into the phylogenetic relationships among wild onions (*Allium*, Amaryllidaceae), with special emphasis on the subgenera *Anguinum* and *Rhizirideum*, as revealed by plastomes

**DOI:** 10.3389/fpls.2023.1124277

**Published:** 2023-03-21

**Authors:** JiYoung Yang, Seon-Hee Kim, Hee-Young Gil, Hyeok-Jae Choi, Seung-Chul Kim

**Affiliations:** ^1^ Research Institute for Dok-do and Ulleung-do Island, Kyungpook National University, Daegu, Republic of Korea; ^2^ Department of Botany, Graduate School of Science, Kyoto University, Kyoto, Japan; ^3^ Division of Forest Biodiversity, Korea National Arboretum, Pocheon, Republic of Korea; ^4^ Department of Biology and Chemistry, Changwon National University, Changwon, Republic of Korea; ^5^ Department of Biological Sciences, Sungkyunkwan University, Suwon, Republic of Korea

**Keywords:** *Allium ulleungense*, *Allium dumebuchum*, Ulleung Island, plastome evolution, infrageneric classification

## Abstract

The genus *Allium*, with over 900 species, is one of the largest monocotyledonous genera and is widely accepted with 15 recognized subgenera and 72 sections. The robust subgeneric and sectional relationships within *Allium* have long been not resolved. Based on 76 species of *Allium* (a total of 84 accessions), we developed a highly resolved plastome phylogenetic framework by integrating 18 newly sequenced species (20 accessions) in this study and assessed their subgeneric and sectional relationships, with special emphasis on the two subgenera *Anguinum* and *Rhizirideum*. We retrieved the three major evolutionary lines within *Allium* and found that the two subgenera *Anguinum* and *Rhizirideum* are monophyletic whereas others are highly polyphyletic (e.g., *Allium*, *Cepa*, *Polyprason*, and *Melanocrommyum*). Within the subgenus *Anguinum*, two strongly supported sublineages in East Asian and Eurasian-American were found. *Allium tricoccum* in North America belonged to the Eurasian clade. The distinct taxonomic status of *A. ulleungense* and its sister taxon were further determined. In subg. *Rhizirideum*, the Ulleung Island endemic *A. dumebuchum* shared its most recent common ancestor with the species from Mongolia and the narrow Korean endemic *A. minus*. Two Ulleung Island endemics were estimated to originate independently during the Pleistocene. In addition, a separate monotypic sectional treatment of the east Asian *A. macrostemon* (subg. *Allium*) and sister relationship between *A. condensatum* and *A. chinense* was suggested.

## 1 Introduction

The genus *Allium* L. is one of the most diverse groups in petaloid monocotyledons, with more than 900 species, and some of them are used medicinally or have economic (e.g., onions, garlic, chives, scallions, leeks, shallots) and horticultural values (Fritsch and Friesen, 2002; Fritsch et al., 2010; Li et al., 2010; Herden et al., 2016). The genus has its main center of diversity in southwest and central Asia and a smaller one in North America, and it is characterized by having bulbus enclosed in membranous tunics, free or almost free tepals, and a usual subgynobasic style (Friesen et al., 2006). Although *Allium* was previously placed in Alliaceae (subfamily Allioideae Herb.) (Fay and Chase, 1996) in the order Amaryllidales (Takhtajan, 1987; Takhtajan, 1997), it is currently recognized as a member of the family Amaryllidaceae in the subfamily Allioideae, which consists of *Allium* only (including *Caloscordum* Herb., *Milula* Prain, and *Nectaroscordum* Lindl.) (Friesen et al., 2006). The infrageneric classification of *Allium* has been varied and complicated because of its size and taxonomic complexity, but the widely accepted and recent one includes 15 subgenera and 72 sections (Friesen et al., 2006). Based on molecular phylogenetic analyses of chloroplast and nuclear DNA sequences, the monophyly of *Allium* has been confirmed, and 15 subgenera and 72 sections have been grouped into three evolutionary lines (Friesen et al., 2006; Li et al., 2010; Wheeler et al., 2013).

**Figure 1:**
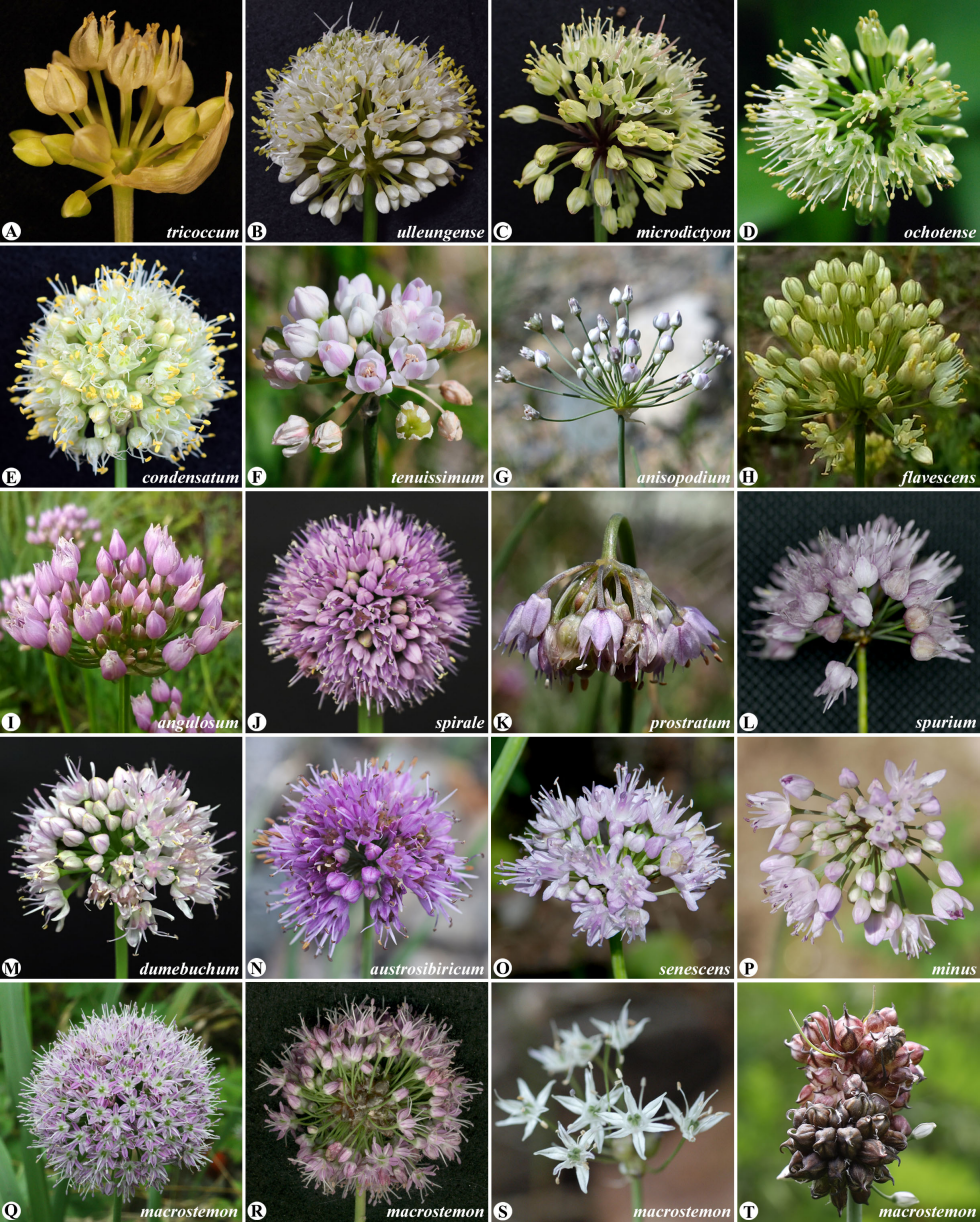
Species of *Allium*, especially from the two subgenera *Anguinum* and *Rhizirideum*, newly sequenced for their plastomes in this study. Subg. *Anguimum*: *A tricoccum* **(A)**, *A ulleungense* **(B)**, *A microdictyon* **(C)**, and *A ochotense* **(D)**. Subg. *Cepa*: *A condensatum* **(E)**. Subg. *Rhizirideum*: *A tenuissimum* **(F)**, *A anisopodium* **(G)**, *A flavescens* **(H)**, *A angulosum* **(I)**, *A spirale* **(J)**, *A prostratum* **(K)**, *A spurium* **(L)**, *A dumebuchum* **(M)**, *A austrosibiricum* **(N)**, *A senescens* **(O)**, and *A minus* **(P)**. Subg. *Allium*: *A macrostemon* **(Q-T)** show floral variations. Photo credit: Hyeok-Jae Choi.

Of the 15 subgenera, subg. *Anguinum* (G. Don. ex W.D.J. Koch) N. Friesen, which consists of ten and several varieties, shows disjunct distribution in high mountains from South West Europe to East Asia and in Northeastern North America (Fritsch and Friesen, 2002) (**Figures 1A–D**) (also see geographical distribution map of subg. *Anguinum* in Figures 3 and 4 of Herden et al., 2016). It is one of the strongly supported monophyletic groups and represents a distinct and specialized group, and especially it is adapted to the light regime under deciduous forests within the second evolutionary line (Pistrick, 1992; Li et al., 2010). Subg. *Anguinum* is characterized by various important characteristics, such as root anatomical traits (Fritsch, 1992a), leaf and bulb structure (Pastor and Valdes, 1985), hypogeal seed germination and seedling type (Druselmann, 1992), and locule and nectary structure (Fritsch, 1992b) and shares the basic chromosome number (x = 8) and karyotype (Jing et al., 1999). For the phylogenetic position of subg. *Anguinum* in the second evolutionary line, previous studies suggested that *Anguinum* is closely related to subg. *Caloscordum* (Herb.) R.M. Fritsch (Nguyen et al., 2008; Li et al., 2010; Herden et al., 2016). Within subg. *Anguinum*, two major lineages exist: the Eurasian-American *Allium victorialis* L. alliance, such as *A. tricoccum* Solander, and the East Asian alliance of *A. prattii* C.H. Wright, *A. ovalifolium* Hand.-Mazz., and others (Friesen et al., 2006; Herden et al., 2016). Although the two major lineages within this subgenus were supported based on nuclear rDNA and three non-coding chloroplast DNA sequences, conflicting nuclear and chloroplast phylogenies suggested potential hybrid origin for *A. tricoccum* in North America (Herden et al., 2016; Choi et al., 2019). The phylogenetic position of *A. tricoccum* has an implication in correctly understanding the intercontinental disjunct distribution *via* possible migration routes between the Bering Land Bridge (BLB) and the North Atlantic Land Bridge (NALB) (Tiffney, 1985; Tiffney and Manchester, 2001).

Another subg. *Rhizirideum* (G. Don ex Koch) Wendelbo, representing one lineage within the third evolutionary line of *Allium*, comprised approximately 37 species placed in five sections (*Rhizirideum*, *Caespitosoprason*, *Tenuissima*, *Rhizomatosa*, and *Eduardia*) (Friesen et al., 2006) (**Figures 1F–P**) (also see geographical distribution map of sect. *Rhizirideum* in Figure 1 of Sinitsyna et al., 2016 and Figure 5 of Jang et al., 2021). These species typically occur in Eurasian steppes, with the greatest diversity in southern Siberia and Mongolia, whereas only a few species are distributed in Europe and East Asia (Korea, Russian Far East, and Japan). Of the five sections, sect. *Rhizirideum*, represents the most speciose group with 26 species, including the recently published new species *A. dumebuchum* from Korea (Jang et al., 2021) and *A. heterophyllum* from China (Xie et al., 2022), which widely occur from Europe to East Asia. Most species are distributed in temperate Asia, whereas only four species occur in Europe, and just two species commonly occur in Europe and reach Western Siberia (Sinitsyna et al., 2016). The species of sect. *Rhizirideum* share a basic chromosome number of x = 8, with various ploidy levels (2x, 4x, 5x, and 6x), potentially suggesting the important role of polyploidization process (Friesen, 1988; Sinitsyna et al., 2016 and references therein). Section *Rhizirideum* appears to be monophyletic, but phylogenetic relationships among species within sect. *Rhizirideum* has been poorly resolved based on traditional markers, such as nrDNA ITS and non-coding chloroplast DNA sequences (Sinitsyna et al., 2016; Jang et al., 2021; Xie et al., 2022).

An infrageneric placement of certain species and section has also been problematic. For example, the phylogenetic position of *A. macrostemon* (**Figures 1Q–T**), a wild onion widespread in East Asia (China, Japan, Korea, Mongolia, Tibet, and Russian Far East), has been disputed. In the taxonomic synopsis of the genus *Allium* in China, *A. macrostemon* was placed in subg. *Allium* sect. *Allium*, along two other species (*A. porrum* and *A. sativum*), but it was traditionally included in subg. *Allium* sect. *Scorodon* sensu lato (Hanelt, 1992). Friesen et al. (2006) placed it under *Allium* or suggested placing it to a new section. Based on the combined nuclear ITS and chloroplast *rps16* sequence analysis of primarily Chinese *Allium* species, *A. macrostemon* was sister to *A. caeruleum* (subg. *Allium* sect. *Caerulea*) (Li et al., 2010). Most recently, the ITS sequence phylogeny showed that the clade containing several accessions of *A. macrostemon* was sister to *A. caeruleum* and that *A. schoenoprasoides* was sister to the *A. macrostemon*-*A. caeruleum* clade (Xie et al., 2022). In contrast, three cpDNA region sequences (*ndhJ*-*trnF*, *psbD*-*trnT*, and *psbJ*-*petA*) indicated a sister relationship between *A. macrostemon* and *A. schoenoprasoides* (Xie et al., 2022). The complete plastome sequence of *A. macrostemon* was reported, and the phylogeny suggested that it is sister to the clade of *A. cepa*–*A. fistulosum*–*A. altaicum* (subg. *Cepa* sect. *Cepa*) (Xie F.-M. et al., 2019). It was suggested that *A. macrostemon* shared similar testa sculptures and morphological characters (i.e., bulbil development in inflorescence, pistil morphology, and seed shape) with members of sect. *Caerulea*, especially with *A. caeruleum* (Choi et al., 2012). However, the membranous bulb tunics, widely spreading and pinkish perianth, and subulate inner filaments do not fit the description of the sect. *Caerulea*; hence, its placement in that section could not be justified (Choi and Oh, 2011). In addition, *A. macrostemon* has been consistently distinguished from its related sections of *Allium* subg. *Allium*, warranting the establishment of a new section (Friesen et al., 2006; Li et al., 2010; Xie et al., 2022). Thus, it is necessary to reassess the phylogenetic position of *A. macrostemon*, with additional sampling, in a much broader plastome phylogenetic framework.

*Allium condensatum* (**Figure 1E**), a wild onion with pale yellow perianth commonly occurring in East Asia (China, Mongol, Korea, and Russian Far East), was placed newly in the monotypic sect. *Condensatum* (subg. *Cepa*) from its original placement in sect. *Oreiprason* F. Herm. (subg. *Polyprason* Radic) (Friesen et al., 2006; Choi et al., 2012). It was shown, based on testa sculpture, that *A. condensatum* in sect. *Condensatum* is clearly distinguished from the other members of sect. *Oreiprason* (e.g., *A. hymenorrhizum* Ledeb. and *A. obliquum* L.) and subg. *Cepa*, supporting its own sectional treatment (Friesen et al., 2006; Choi et al., 2012). However, previous studies suggested that *A. condensatum* is closely related to species of subg. *Allium* sect. *Codonoprason* Rschb., e.g., *A. carinatum* L., *A. flavum* L., and *A. melanantherum* Pancic (Kruse, 1984). Therefore, it has been uncertain whether *A. condensatum* requires its own sectional treatment or it should belong to subg. *Allium*, *Polyprason*, or *Cepa*. There is a need to evaluate the phylogenetic position of *A. condensatum* (sect. *Condensatum*) within the up-to-date *Allium* plastome phylogenetic framework.

Although concatenated chloroplast non-coding region sequences had limited values, complete plastome sequences have been treasured and used recently to reveal the evolutionary history and adaptive evolution of the genus *Allium* and related genera (Huo et al., 2019; Xie D.-F. et al., 2019, Xie et al., 2020; Yang et al., 2020; Namgung et al., 2021; Scobeyeva et al., 2021; Chen et al., 2022; Jin et al., 2022). In particular, a total of 39 complete plastomes of *Allium*, covering 12 subgenera, provided well-supported phylogenetic relationships, identified numerous positively selected genes, and confirmed the monophyly of the genus and the three evolutionary lines (Xie et al., 2020). Furthermore, new species relationships were revealed within the poorly resolved third evolutionary lineage. Recently, nine plastomes of *Allium* species were assembled and, based on 38 species of *Allium* and the 11 other Amaryllidaceae species, the functionality loss of *rps16*, *rps2*, *infA*, and *ccsA* genes was documented within the genus (Scobeyeva et al., 2021).

As an ongoing effort to better understand the fascinating evolutionary history among species of *Allium* in Korea and neighboring countries in East Asia, we characterized their chloroplast genomes and conducted population genetics and phylogeographic investigations. In this study, we newly sequenced 11 species of *Allium* from subg. *Rhizirideum* and seven accessions, representing five species of *Allium* from subg. *Anguinum*, and analyzed them within the most comprehensive *Allium* plastome framework (a total of 84 accessions representing 76 species). We also sequenced two additional species, *A. macrostemon* and *A. condensatum*, to assess their phylogenetic positions. The aims of this study were (1) to reevaluate the infrageneric classification of the genus *Allium* based on the most comprehensive plastome framework, (2) to characterize additional plastome sequences of *Allium* species primarily from two subgenera, *Anguinum* and *Rhizirideum*, (3) to determine species relationships within *Anguinum* and *Rhizirideum*, with special emphasis on newly described species in Korea, and (4) to assess the phylogenetic position of *A. macrostemon* and *A. condensatum* (sect. *Condensatum*).

## 2 Materials and methods

### 2.1 Plant materials

For subg. *Anguinum*, we sampled a total of seven accessions, representing five species: one accession of *A. tricoccum*, two accessions of *A. ochotense*, two accessions of *A. victorialis*, one accession of *A. ulleungense*, and one accession of *A. microdictyon* (see complete species list in **Table 1**). Two accessions of previously reported plastome sequences under the names of *A. victorialis* (NC037240) and *A. ochotense* (NC057853) from Ulleung Island, Korea, are considered *A. ulleungense* sequenced in this study (**Table 1**). Two accessions of *A. victorialis* were sampled from Austria and Germany, and two accessions of *A. ochotense* were sampled from China and Japan. We sampled *A. microdictyon* from Sobaeksan Mountain in Korea. For subg. *Rhizirideum*, we sampled a total of 11 accessions, representing two sections of *Rhizirideum*: sect. *Tenuissima* (*A. anisopodium* and *A. tenuissimum*) and sect. *Rhizirideum* (*A. flavescens*, *A. angulosum*, *A. senescens*, *A. spirale*, *A. dumebuchum*, *A. spurium*, *A. prostratum*, *A. austrosibiricum*, and *A. minus*). Lastly, we sampled one representative of subg. *Cepa* sect. *Condensatum* (*A. condensatum*) from Russian Far East and one representative of subg. *Allium* sect. *Allium* (*A. macrostemon*) from Ulleung Island, Korea.

**Table 1:** Summary of the characteristics of the 20 *Allium* chloroplast genomes.

Taxa	Total cpDNA size (bp)	GC content (%)	LSC size (bp) /GC content (%)	IR size (bp) /GC content (%)	SSC size (bp) /GC content (%)	No. of genes	No. of protein-coding genes	No. of tRNA genes	No. of rRNA genes	No. of duplicated genes	Accession number
***A. angulosum***	153,473	36.9	82,553/34.7	26,487/42.7	17,946/29.6	132	85	38	8	19	OP743930
***A. anisopodium***	153,218	36.9	82,502/34.7	26,492/42.7	17,732/29.7	132	84	38	8	19	OP754898
***A. austrosibiricum***	153,493	36.8	82,533/34.7	26,491/42.7	17,978/29.6	132	85	38	8	19	OP743931
***A. condensatum***	153,195	36.8	82,107/34.6	26,505/42.7	18,078/29.5	132	85	38	8	19	OP743932
***A. dumebuchum***	153,507	36.8	82,549/34.7	26,491/42.7	17,976/29.6	132	85	38	8	19	OP743933
***A. flavescens***	153,428	36.9	82,482/34.7	26,487/42.7	17,972/29.6	132	85	38	8	19	OP743934
***A. macrostemon***	153,126	36.8	82,049/34.6	26,499/42.7	18,079/29.3	132	83	38	8	19	OP743935
***A. microdictyon*** (Korea)	153,561	37.0	82,621/34.9	26,542/42.7	17,845/29.9	132	85	38	8	19	OP743936
***A. minus***	153,499	36.8	82,538/34.7	26,491/42.7	17,979/29.6	132	85	38	8	19	OP743937
***A. ochotense*** (China)	153,544	37.0	82,605/34.9	26,542/42.7	17,855/29.9	132	85	38	8	19	OP743938
***A. ochotense*** (Japan)	153,121	37.0	82,180/34.9	26,542/42.7	17,857/29.9	132	85	38	8	20	OP743939
***A. prostratum***	153,597	36.8	82,630/34.7	26,371/42.7	18,225/29.5	132	85	38	8	19	OP743940
***A. senescens***	153,490	36.8	82,529/34.7	26,491/42.7	17,979/29.6	132	85	38	8	19	OP743941
***A. spirale***	153,584	36.8	82,585/34.7	26,504/42.7	17,991/29.5	132	85	38	8	19	OP743942
***A. spurium***	153,529	36.8	82,563/34.7	26,492/42.7	17,982/29.5	132	85	38	8	19	OP743943
***A. tenuissimum***	153,251	36.9	82,408/34.7	26,381/42.7	17,971/29.7	132	85	38	8	19	OP743944
***A. tricoccum***	153,589	37.1	82.595/35.0	26,572/42.7	17,850/30.0	132	84	38	8	19	OP743945
***A. ulleungense***	154,049	37.0	83,145/34.9	26,526/42.7	17,852/30.0	132	85	38	8	19	OP743946
***A. victorialis*** (Austria)	153,652	37.0	82,679/34.9	26,548/42.7	17,877/29.9	132	85	38	8	19	OP743947
***A. victorialis*** (Germany)	153,361	37.0	82,918/34.9	26,217/42.8	18,009/29.9	132	84	38	8	19	OP743948

### 2.2 DNA isolation, NGS sequencing, and comparative plastome analysis

Fresh leaves were collected and dried using silica gel, and the total genomic DNA was extracted using DNeasy Plant Mini Kit (Qiagen, Carlsbad, CA, USA). The extracted DNA was sequenced using an Illumina HiSeq 4000 (Illumina, Inc., San Diego, CA, USA) at Macrogen Co. (Seoul, Korea), and it yielded a 150-bp paired-end read length. The resulting paired-end reads were assembled *de novo* using Velvet v1.2.10 with multiple k-mers (Zerbino and Birney, 2008). The complete plastomes were also confirmed by NOVOPlasty v2.6.2. (Dierckxens et al., 2016) using *A. cepa* (MK335926) and *A. sativum* (MK335928) as references. tRNAs were confirmed using tRNA scan-SE (Lowe and Eddy, 1997), and the sequences were annotated using Geneious R10 (Kearse et al., 2012). Annotated sequence files in the GenBank format were used to draw a circular map using OGDRAW v1.2 (Greiner et al., 2019). We used DnaSP v6.10 (Rozas et al., 2017) to perform a sliding window analysis with a step size of 200 bp and a window length of 800 bp to determine the nucleotide diversity (Pi) of the plastomes.

### 2.3 Phylogenetic analysis and molecular dating

The complete plastome sequences were aligned using MAFFT v7 (Katoh and Standley, 2013), and a maximum likelihood (ML) phylogenetic tree was constructed using IQ-TREE, with 1,000 bootstrap replicates (Nguyen et al., 2015). The aligned sequences in FASTA format are available in **Supplementary Data Sheet 1**. The best-fit evolutionary model for the complete plastome sequences, TVM+F+R6, was selected based on ModelFinder (Kalyaanamoorthy et al., 2017) implemented in IQ-TREE v1.4.2. We used *Agapanthus* as an outgroup based on a previous study (Xie et al., 2020). We also performed maximum parsimony (MP) and Bayesian inference (BI) analysis to evaluate tree topology based on different phylogenetic methods. For MP analysis, Fitch parsimony was performed with PAUP*4.0b10 (Swofford, 2003) using the HEURISTIC search option with TBR branch swapping and MULPARS on. Gaps were treated as missing, and bootstrap support for groups were determined by 1,000 bootstrap replicates (Felsenstein, 1985) using the HEURISTIC search option from a simple addition sequence with TBR branch swapping. To construct a BI tree, we used the MrBayes v3.2.6 (Ronquist et al., 2012) based on the best-fit model (GTR+G) from MrModeltest v2.2 (Nylander, 2004). The analysis was performed with one million generations initiated with a random starting tree, sampling every 1,000 generations. Tracer v1.7.1 (Rambaut et al., 2018) was used to evaluate the burn-in and to examine log likelihoods, ensuring that the run was in the stationary phase and that adequate effective sample sizes (ESS) were attained. After discarding initial 25% as burn-in, the remaining samples were used to construct a 50% majority-consensus tree with posterior probabilities (PP) for given clades. The consensus tree was finally edited using FigTree v1.4.3. (available online: http://tree.bio.ed.ac.uk/software/giftree/).

Divergence times based on the complete chloroplast genome sequences were estimated using the Bayesian method (Drummond et al., 2006) using the program BEAST v1.10.4 (Suchard et al., 2018). The XML file for the analysis was prepared in the Bayesian Evolutionary Analysis Utility (BEAUTi). As the secondary calibration point, we used the crown *Allium* clade mean age of 22.18 Myr and standard deviation of 9.93, resulting in a range of 15.23–34.81 Myr (Xie et al., 2020). We used the Yule process speciation prior, a lognormal relaxed clock model, and GTR+G substitution model, and then the ucld. mean parameter was specified to be uniform with 0.333 as the initial value, 0.00 as the lower, and 1 as the upper limit (Drummond et al., 2006). Posterior distributions for each parameter were estimated by means of an MCMC run for 30 million generations with a sampling frequency of every 100,000 generations. The posterior distribution of all statistics was checked using Tracer v1.7.1 (Rambaut et al., 2018) to assess convergence and confirm that the effective sample sizes (ESS) for all parameters were larger than 200 (Drummond et al., 2012). In addition, we used TreeAnnotator version 1.5 (http://beast.bio.ed.ac.uk/TreeAnnotator) to produce a maximum credibility tree of mean divergence time and 95% highest posterior density (HPD) intervals with posterior probability (PP) limit (0.5), after removing the first 25% of trees as burn-in (Drummond et al., 2012).

## 3 Results

### 3.1 Plastome characteristics of two subgenera *Rhizirideum* and *Anguinum*

**Figure 2:**
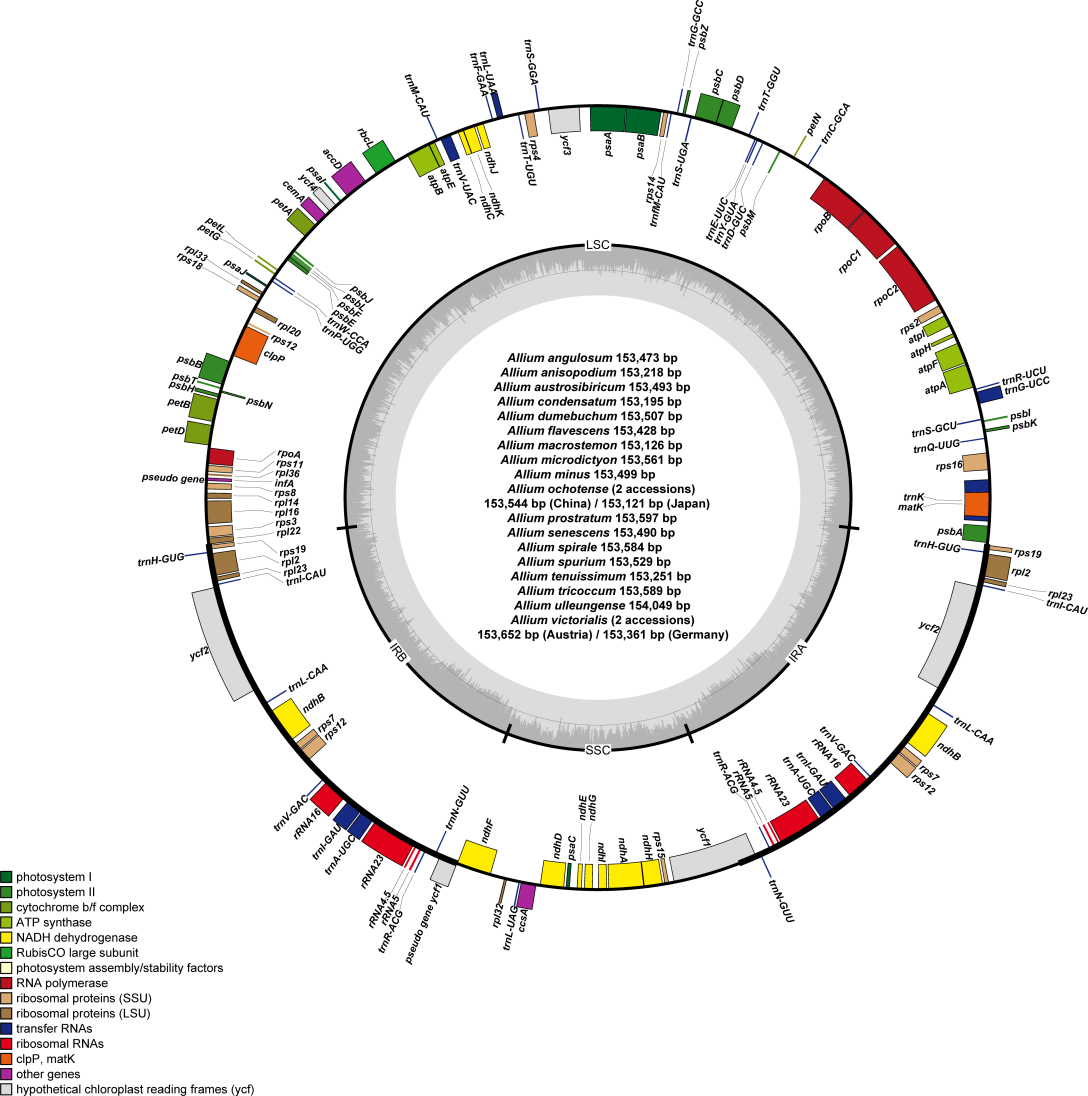
Complete plastome map of newly sequenced *Allium* species (a total of 20 accessions from 18 species) sequenced in this study. The genes inside and outside of the circle are transcribed in the clockwise and counterclockwise directions, respectively. Genes belonging to different functional groups are shown in different colors. The thick lines indicate the extent of the inverted repeats (IRa and IRb) that separate the genomes into small single copy (SSC) and large single copy (LSC) regions.

The plastomes of 11 newly sequenced *Allium* accessions from subg. *Rhizirideum* were 153,218 (*A. anisopodium*) to 153,597 bp (*A. prostratum*) in length and comprised a large single copy (LSC) region of 82,408 (*A. tenuissimum*) to 82,630 bp (*A. prostratum*), a small single copy (SSC) region of 17,732 (*A. anisopodium*) to 18,225 bp (*A. prostratum*), and two inverted repeat (IR) regions of 26,371 (*A. prostratum*) to 26,504 bp (*A. spirale*) (**Table 1** and **Figure 2**). The overall guanine–cytosine (GC) content ranged from 36.8% (*A. austrosibiricum*, *A. dumebuchum*, *A. minus*, *A. prostratum*, *A. senescens*, *A. spirale*, and *A. spurium*) to 36.9% (*A. angulosum*, *A. anisopodium*, and *A. tenuissimum*). One species, *A. anisopodium*, had 84 protein-coding genes with two pseudogenized *ycf1* genes, and a shortened sequence of 5,248 bp of *ycf1* gene in the IR region at the SSC/IRa junction became a pseudogene. Furthermore, a completely functional *rps2* gene was observed in all except one species (*A. spurium*) in subg. *Rhizirideum*, which contrasts with most *Allium* species in the third evolutionary line showing pseudogenization.

The plastomes of seven newly sequenced *Allium* accessions from subg. *Anguinum* were 153,121 bp (*A. ochotense*, Japan), 153,361 bp (*A. victorialis*, Germany), 153,544 bp (*A. ochotense*, China), 153,561 bp (*A. microdictyon*), 153,589 bp (*A. tricoccum*), 153,652 bp (*A. victorialis*, Austria), and 154,049 bp (*A. ulleungense*) in length. The overall guanine–cytosine (GC) content was 37% identical within subg. *Anguinum*, except for *A. tricoccum* with 37.1% GC content. *Allium victorialis* sampled from Germany and *A*. *tricoccum* contained 84 protein coding genes with two pseudogenized *ycf1* genes: the shortened sequences of 4,254 bp (*A. tricoccum*) and 4,161 bp (*A. victorialis*) were in the IR region at the SSC/IRa junction and have become a pseudogene. Like most *Allium* species in the second evolutionary line, two functional genes, *rps2* and *rps16*, were found in all subg. *Anguinum* species.

### 3.2 Plastome characteristics of *A. macrostemon* and *A. condensatum*

To further explore the phylogenetic position of *A. macrostemon*, we sequenced one additional accession sampled from Ulleung Island, Korea. The complete plastome was 153,126 bp in length with an overall GC content of 36.8% and comprised a large single copy (LSC) region of 82,049 bp with 34.6% GC content, a small single copy (SSC) region of 18,079 bp with 29.3% GC content, and two inverted repeat (IR) regions of 26,499 bp with 42.7% GC content (**Table 1**). We observed that *rps2* and *rps16* genes had become a pseudogene in *A. macrostemon*. Thus, a total of 83 protein-coding genes were detected in this species and the remaining *Allium* plastomes contained a total of 85 protein-coding genes. The complete plastome of *A. condensatum* sampled from Russia was 153,195 bp in length with an overall GC content of 36.8% and comprised a large single copy (LSC) region of 82,107 bp with a 34.6% GC content, a small single copy (SSC) region of 18,078 bp with a 29.5% GC content, and two inverted repeat (IR) regions of 26,505 bp with a 42.7% GC content. The *rps2* gene was shortened in this species, whereas the *rps16* gene was a completely functional gene with a completely conserved domain.

### 3.3 Comparative analysis of plastome structure and mutation hotspots in genus *Allium*

The position of IR/SC borders were determined in the 84 *Allium* plastomes, representing the three major evolutionary lines (**Supplementary Figure 1**). We found gene contents on both sides of the IR/SC borders conserved, and the LSC/IRb border was *rps19*/*rpl22*, whereas the IRa/LSC border was *rps19*/*psbA*. While the IR/SC borders among the species of *Allium* in the first evolutionary line (four subgenera *Nectaroscordum*, *Microscordum*, *Amerallium*, and *Cyathophora*) are conserved, some exceptions included the following: (1) the *rpl22* gene of *A. montanum* (subg. *Microscordum*) and *A. kingdonii* (subg. *Cyathophora*) entirely in the LSC region; (2) the *ndhF* gene entirely in the SSC region for *A. siculum* (subg. *Nectaroscordum*), *A. macranthum* (subg. *Amerallium*), *A. kingdonii*, and *A. zebdanense* (subg. *Amerallium*); and (3) the *rps19* gene of *A. kindgonii* in the IRb/LSC border (**Supplementary Figure 1**). Within the second evolutionary line of *Allium* (three subgenera, *Caloscordum*, *Melanocrommyum*, and *Anguinum*), some exceptions included the following: (1) the *rpl22* gene of subg. *Melanocrommyum* (*A. fetisowii*, *A. macleanii*, and *A. karataviense*) and *A. victorialis*, Germany OP743948 (subg. *Anguinum*), completely in the LSC region; (2) the *ndhF* gene completely in the SSC region for subg *Caloscordum* and *Melanocrommyum*, and subg. *Anguinum* (*A. ovalifolium* var. *leuconeurum* and *A. victorialis*, Germany, OP743948); and (3) the *rps19* gene in the IRb/LSC border for *A. fetisowii* (subg. *Melanocrommyum*) and *A. victorialis*, Germany OP743948 (subg. *Anguinum*) (**Supplementary Figure 1**). Lastly, the third evolutionary line included eight subgenera, *Butomissa*, *Cyathophora*, *Allium*, *Cepa*, *Reticulatobulbosa*, *Rhizirideum*, *Melanocrommyum*, and *Polyprason*. We found that the *rpl22* gene was entirely located in the SSC region for subgenera *Cyathophora*, *Rhizirideum* (*A. mongolicum*, *A. tenuissimum*, and *A. anisopodium*), *Allium* (*A. caeruleum*), and *Polyprason* (*A. xichuanense*, *A. rude*, and *A. chrysanthum*). The *rps19* gene of subg. *Cyathophora* (*A. mairei* and *A. spicatum*) and subg. *Rhizirideum* (*A. mongolicum* and *A. tenuissimum*) bordered in IRb/LSC. The *rps19* gene of *A. tenuissimum* (subg. *Rhizirideum*) and *A. mairei* (subg. *Cyathophora*) bordered in IRa/LSC.

**Figure 3:**
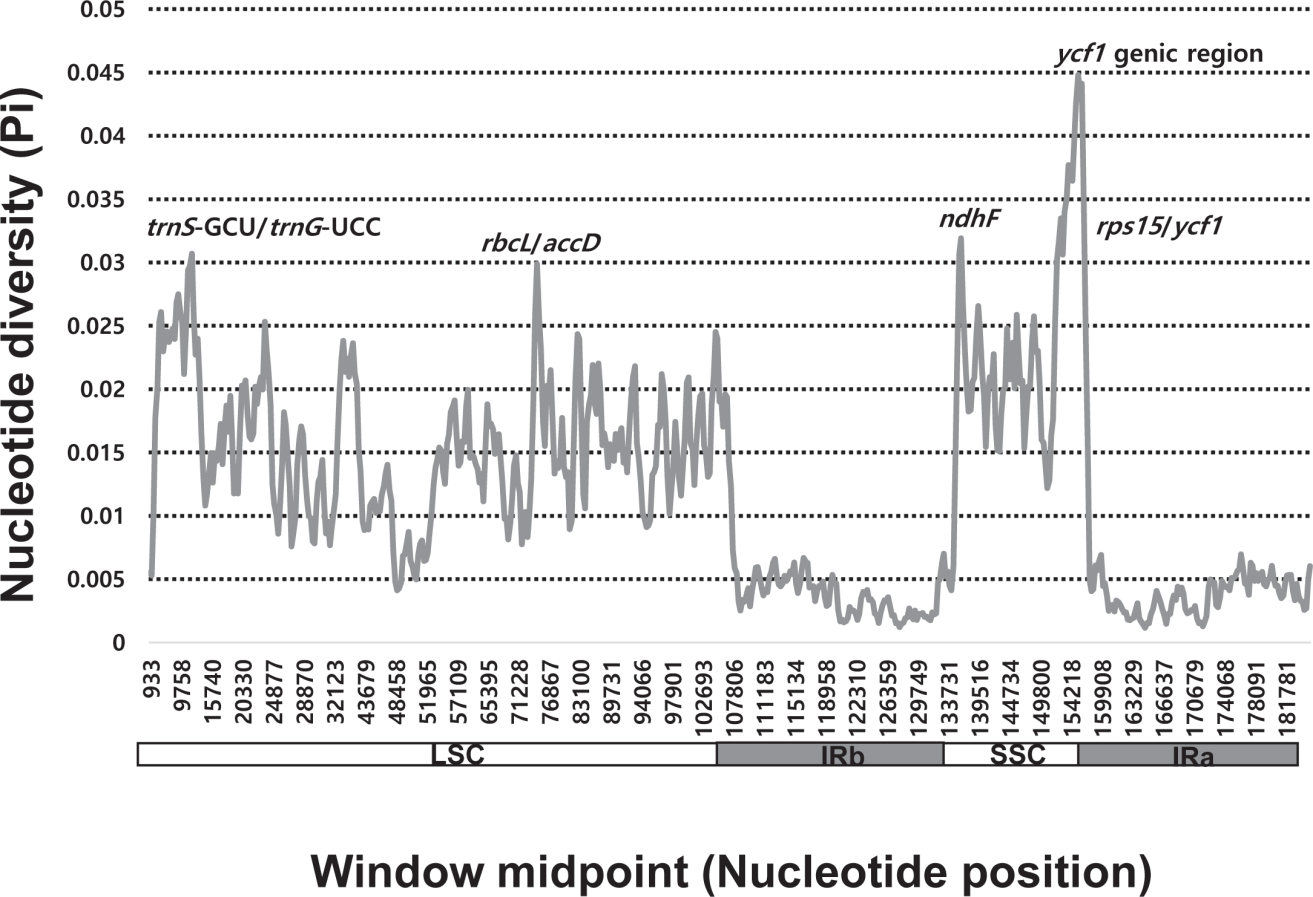
Five most variable regions found among the three evolutionary lines of genus *Allium*.

Sliding window analysis revealed highly variable regions in the plastomes of 84 *Allium* accessions representing the three evolutionary lines (**Figure 3**). The average nucleotide diversity (Pi) over the entire plastomes was 0.01191, with the most variable region (Pi = 0.0448) being the *ycf1* genic region followed by the *ndhF* genic region (Pi = 0.03192). Three additional intergenic regions, i.e., *trnS*-GCU/*trnG*-UCC (Pi = 0.03068), *rps15*/*ycf1* (Pi = 0.03018), and *rbcL*/*accD* (Pi = 0.02988), were also highly variable.

### 3.4 Phylogenetic analysis

**Figure 4:**
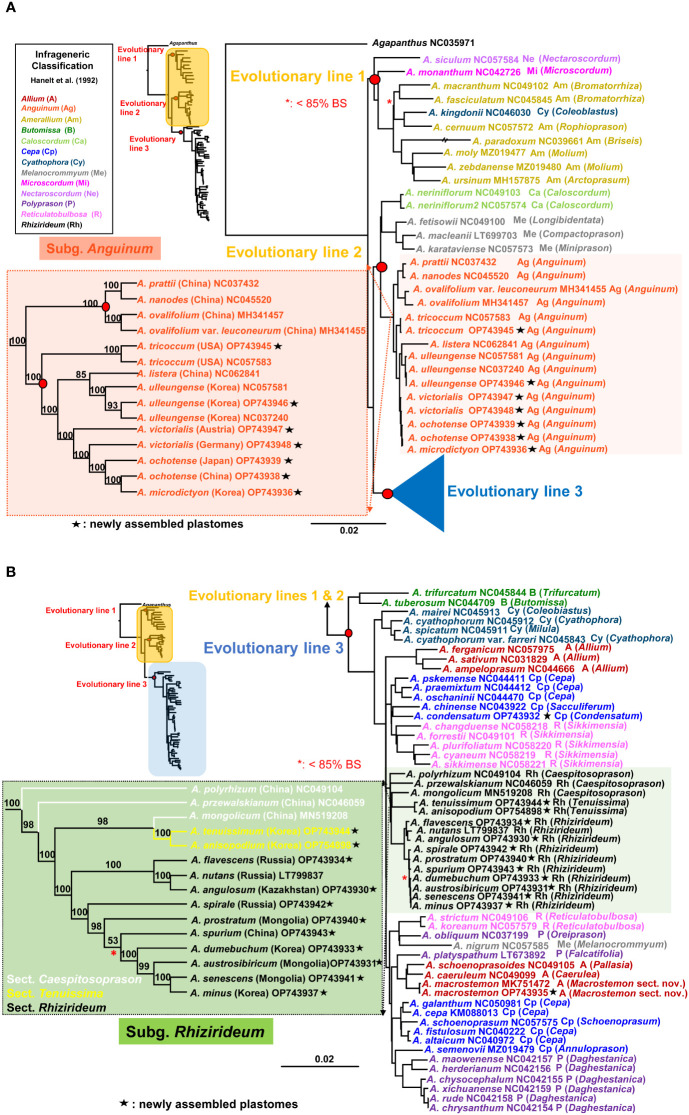
Maximum likelihood (ML) tree of the genus *Allium* based on 84 accessions representing 76 species. **(A)** A partial ML tree showing the first and second evolutionary lines of *Allium*, with enlargement of subg. *Anguinum*. Node with < 85% bootstrap support (BS) is shown in red asterisk. **(B)** A partial ML tree showing the third evolutionary line of *Allium*, with enlargement of subg. *Rhizirideum*. Node with < 85% bootstrap support (BS) is shown in red asterisk. Newly assembled plastome in this study is shown in black asterisk. Subgenera (Hanelt et al., 1992) are indicated in different colored labels. A species name is followed by a NCBI accession number, a subgeneric abbreviation, and a section in parenthesis.

The most comprehensive plastome sequences (a total of 84 accessions representing 76 species) provided a well-resolved and highly supported ML phylogenetic framework of the genus *Allium* (**Figures 4A, B**). It revealed three major evolutionary lines within the genus and consistently indicated that the first evolutionary line diverged first, followed by the second and third evolutionary lines. Within the first evolutionary line (100% BS), *A. siculum*, which belongs to subg. *Nectaroscordum* (sect. *Nectaroscordum*), diverged first, followed by *A. monanthum* (subg. *Microscordum* sect. *Microscordum*) and the clade of subg. *Amerallium* (100% BS) (**Figure 4A**). *Allium kingdonii* (subg. *Cyathophora*), which occurs very rarely in southeastern Tibet, was deeply embedded within the subg. *Amerallium*: *A. kingdonii* was sister to *A. cernuum* (100% BS), which belongs to sect. *Lophioprason* and the most widespread North American species of the genus. In the case of sectional relationships within subg. *Amerallium*, sect. *Briseis* (*A. paradoxum*) is sister to the clade containing two sections, *Molium* (*A. moly* and *A. zebdanense*) and *Arctoprasum* (*A. ursinum*) (100% BS).

For the second evolutionary line, the plastome phylogeny showed two major evolutionary lineages: one includes subg. *Anguinum* (100% BS) and the other (99% BS) includes two reciprocally monophyletic subgenera, *Melanocrommyum* (100% BS) and *Caloscordum* (100% BS) (**Figure 4A**). Within subg. *Melanocrommyum*, *A. karataviense* (sect. *Miniprason*) was sister to *A. macleanii* (sect. *Compactoprason*) and *A. fetisowii* (sect. *Longibidentata*) was sister to the clade of *A. macleanii*–*A. karataviense* (100% BS). For the species relationship within subg. *Anguinum*, two sublineages were found: one sublineage (100% BS) includes primarily East Asian species (*A. nanodes*, *A. pratii*, *A. ovalifolium*, and *A. ovalifolium* var. *leuconeurum*) whereas the other sublineage (100% BS) includes primarily Eurasian-American species (*A. tricoccum*, *A. ochotense*, *A. victorialis*, *A. ulleungense*, and *A. microdictyon*) and one exceptional species from East Asian sublineage (*A. listera*) (**Figure 4A**). Our result strongly supported that *A. tricoccum* in North America is sister to the clade containing Eurasian species (*A. ochotense*, *A. ulleungense*, *A. victorialis*, and *A. microdictyon*) and one East Asian species (*A. listera*). Both species, *A. victorialis* and *A. ochotense*, appeared not to be monophyletic, and *A. microdictyon* was embedded within *A. ochotense*. One newly sequenced accession of *A. tricoccum* in this study was sister to the other accession previously reported (NC057583). In addition, one newly sequenced accession of *A. ulleungense* (OP743946) in this study formed a clade with other previously reported accessions (NC057583 and NC037240). A distinct species recognition of *A. ulleungense* on Ulleung Island, Korea, is further supported based on the complete plastome sequences in this study: *A. ulleungense* was sister to *A. listera*, which occurs in several provinces in China (Anhui, Hebei, Henan, Jilin, Shaanxi, and Shanxi).

The third most diverse evolutionary line includes several subgenera, such as *Allium*, *Butomissa*, *Cepa*, *Cyathophora*, *Polyprason*, *Rhizirideum*, and *Reticulatobulbosa* (**Figure 4B**). Two monophyletic subgenera *Butomissa* sensu Huang et al. (2014) and *Cyathophora* diverged first and then followed by the diversification of non-monophyletic subgenera *Allium*, *Cepa*, and *Polyprason*. Two lineages of subg. *Allium* were found in this study: one group (*A. ferganicum*, *A. ampeloprasum*, and *A. sativum* from sect. *Allium*; 100% BS) was sister to the remaining clade of the third evolutionary line, whereas the other group of two species (*A. macrostemon* and *A. caeruleum*) was closely related to *A. schoenoprasoides* (subg. *Allium* sect. *Pallasia*) and *A. platyspathum* (subg. *Polyprason* sect. *Falcatifolia*). *Allium macrostemon* sampled from Ulleung Island (Korea; OP743935) in this study and previous accession from China (Jiangsu Province; MK751472) is monophyletic (100% BS) and sister to the clade containing *A. schoenoprasoides* and *A. caeruleum* (100% BS). In the case of subg. *Cepa*, at least four sublineages appeared to exist based on the plastome sequences: (1) *A. chinense* (sect. *Sacculiferum*), which is sister to *A. condensatum* (100% BS), (2) the clade containing sect. *Cepa* (*A. pskemense*, *A. oschaninii*, and *A. praemixtum*; 100% BS), (3) the clade containing other species of sect. *Cepa* (*A. cepa*, *A. galanthum*, *A. altaicum*, and *A. fistulosum*) and sect. *Schoenoprasum* (*A. schoenoprasum*) (100% BS), and (4) *A. semenovii* (sect. *Annuloprason*), which is sister to one clade of subg. *Polyprason* (100% BS) (**Figure 4B**). At least three sublineages of subg. *Polyprason* seemed to exist: (1) a clade containing species in sect. *Daghestanica* (*A. herderianum*, *A. maowenense*, *A. chysocephalum*, *A. xichuanense*, *A. rude*, and *A. chrysanthum*; 100% BS), (2) *A. obliquum* (sect. *Oreiprason*), which is sister to *A. nigrum* (subg. *Melanocrommyum*) (93% BS), and (3) *A. platyspathum* (sect. *Falcatifolia*), which is sister to one subg. *Allium* clade (100% BS). However, subg. *Melanocrommyum* belongs to the second evolutionary line, and *A. nigrum* was unexpectedly placed in the third evolutionary line: it is sister to *A. obliquum* (subg. *Polyprason*). In the case of subg. *Reticulatobulbosa*, two plastome lineages exist, one such as sect. *Sikkimense* (*A. changduense*, *A. forrestii*, *A. plurifoliatum*, *A. cyaneum*, and *A. sikkimense*; 100% BS) and the others such as sect. *Reticulatobulbosa* (*A. strictum* and *A. koreanum*) (100% BS).

As one major lineage within the third evolutionary line, subg. *Rhizirideum* is monophyletic (100% BS) and is sister to the clade containing one small sublineage of *Reticulatobulbosa*, one clade of *Allium*, one clade of *Cepa*, and one major clade of *Polyprason* (84% BS) (**Figure 4B**). Within subg. *Rhizirideum*, sect. *Caespitosoprason* is paraphyletic whereas sect. *Rhizirideum* is monophyletic. Section *Tenuissima* (*A. anisopodium* and *A. tenuissimum*) is monophyletic (100% BS), and *A. mongolicum* (sect. *Caespitosoprason*) is sister to sect. *Tenuissima* (98% BS). Within sect. *Rhizirideum*, two sublineages were identified: one includes *A. flavescens*, *A. angulosum*, and *A. nutans* (100% BS), and the other includes *A. senescens*, *A. spirale*, *A. dumebuchum*, *A. spurium*, *A. prostratum*, *A. austrosibiricum*, and *A. minus* (100% BS). Two endemic species in Korea, *A. minus* and *A. dumebuchum*, are sisters to *A. senescens* and the clade containing *A. austrosibiricum*–*A. senescens*–*A. minus*, respectively (each 100% BS). Lastly, *A. condensatum* is sister to *A. chinense* (subg. *Cepa* sect. *Sacculiferum*) (100% BS).

Of a total of 158,090 aligned characters used for MP analysis, we found 123,356 constant characters (78.03%), 19,806 variable sites (12.52%), and 14,928 parsimony informative characters (9.44%). The heuristic search resulted in four equally most parsimonious trees, with a tree length of 59,095, a consistency index (CI) of 0.70, and a retention index (RI) of 0.82 (**Supplementary Figure 2**). A 50% bootstrap consensus tree was well resolved with high bootstrap support (BS) values and found the same three evolutionary lines identified in ML analysis (**Figure 4**). The MP tree topology was nearly identical to that of ML in phylogenetic relationships among the three evolutionary lines and subgeneric relationships within each evolutionary line. Within subg. *Rhizirideum* and *Anguinum* clade, the species relationships were identical to that of the ML tree, except for the position of *A. listera* in subg. *Anguinum*: *A. listera* is sister to the clade containing *A. ulleungense*–*A. victorialis*–*A. ochotense*–*A. microdictyon* in the MP tree (**Supplementary Figure 2**), whereas it is sister to *A. ulleungense* species only in ML (**Figure 4A**). As with the ML tree (**Figure 4**), we found nearly identical tree topology in the BI tree (**Supplementary Figure 3**), except for the position of *A. nigrum*. Within two subgenera, *Rhizirideum* and *Anguinum*, we found identical tree topology between the ML and BI trees.

### 3.5 Molecular dating

**Figure 5:**
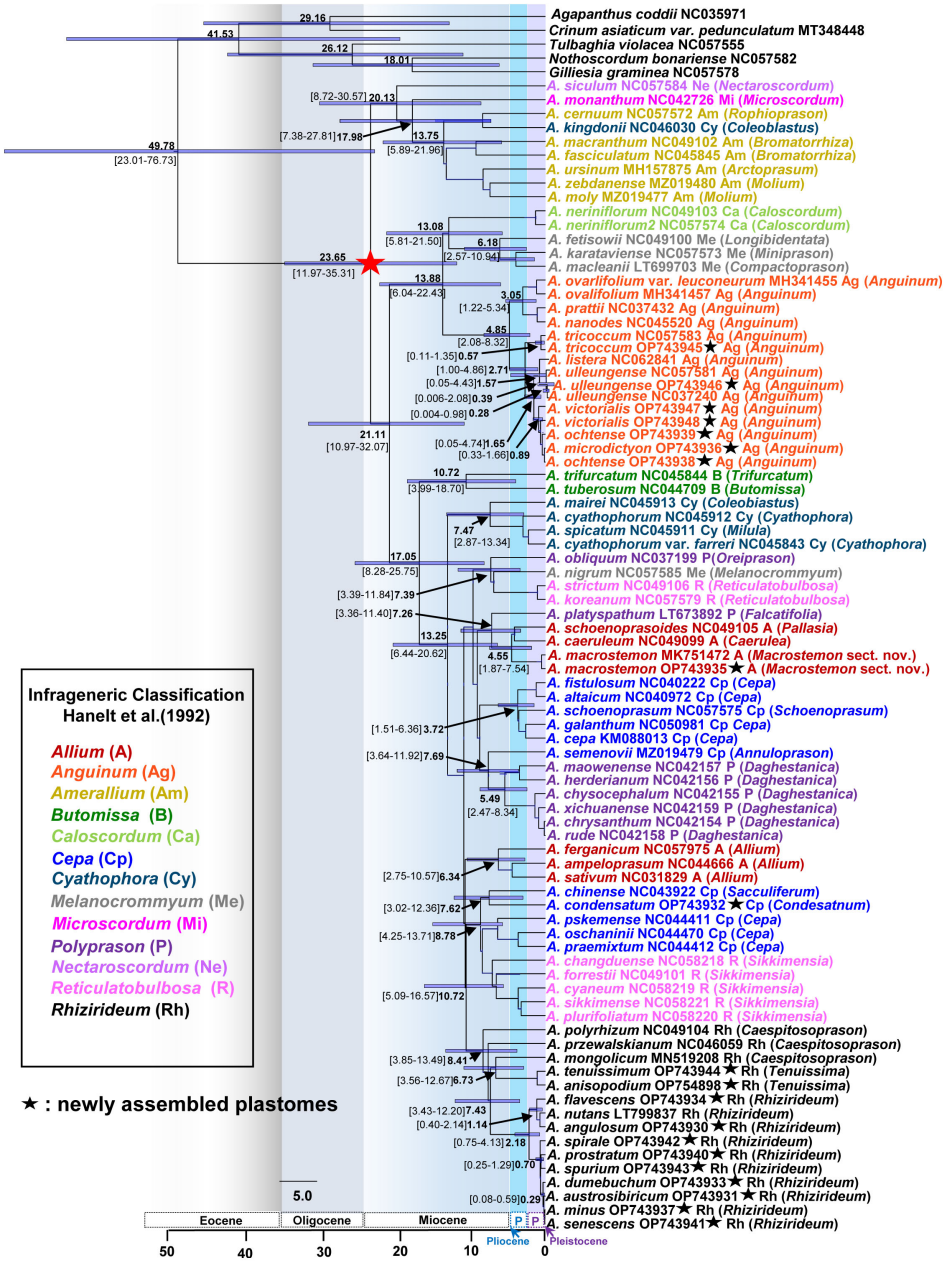
Dated chronogram showing divergence times of major lineages of the genus *Allium*, including three evolutionary lines identified previously. Estimated mean ages are shown in each node with 95% high posterior density (HPD) in bracket. The secondary calibration point estimated previously are shown as asterisked node (red star); that is, the crown age of the genus *Allium* was used in this study. Newly assembled plastome in this study is shown in black asterisk. Subgenera (Hanelt et al., 1992) are indicated in different colored labels. A species name is followed by an NCBI accession number, a subgeneric abbreviation, and a section in parenthesis.

We estimated the divergence times of major lineages within the genus *Allium* and several other lineages of our interests. The crown age of the three evolutionary lines within the genus was estimated to be 20.13 Ma (95% HPD, 8.72–30.57 Ma), 13.88 Ma (95% HPD, 6.04–22.43 Ma), and 17.05 Ma (95% HPD, 8.28–25.75 Ma) for the first, second, and third evolutionary line, respectively, suggesting their origins in early Miocene (**Figure 5**). The crown age of subg. *Anguinum* was estimated to be 4.85 Ma (95% HPD, 2.08–8.32 Ma), whereas that of subg. *Rhizirideum* was 8.41 Ma (95% HPD, 3.85–13.49 Ma). The split of subg. *Anguinum* from its sister lineage (subgenera *Caloscordum* and *Melanocrommyum*) was estimated to be 13.88 Ma (95% HPD, 6.04–22.43 Ma). The subg. *Rhizirideum* was estimated to be a split from two lineages (one lineage of *Cepa* and another lineage of *Reticulatobulbosa*) at 10.72 Ma (95% HPD, 5.09–16.57 Ma). Within subg. *Anguinum*, the crown ages of the two major lineages, representing East Asian and Eurasian-American, were estimated to be 3.05 Ma (95% HPD, 1.22–5.34 Ma) and 2.71 Ma (95% HPD, 1.00–4.86 Ma), respectively. Molecular dating also suggested that the split of Ulleung Island endemic *A. ulleungense* from its sister taxon (*A. listera*) was estimated at 1.57 Ma (95% HPD, 0.05–4.43 Ma). In addition, the intercontinental disjunct event between East Asia and Eastern North America (*A. tricoccum*) was estimated at approximately 2.71 Ma (95% HPD, 1.00–4.86 Ma) in the late Pliocene. Within subg. *Rhizirideum*, the split of monophyletic sect. *Rhizirideum* from two early diverged sections, *Caespitosoprason* and *Tenuissima*, was estimated at 7.43 Ma (95% HPD, 3.43–12.20 Ma). In addition, the split between European (*A. flavescens*–*A. nutans*–*A. angulosum*) and Asian (*A. spirale*–*A. prostratum*–*A. spurium*–*A. dumebuchum*–*A. austrosibiricum*–*A. senescens*–*A. minus*) lineages within sect. *Rhizirideum* was estimated at 2.18 Ma (95% HPD, 0.75–4.13 Ma). *Allium dumebuchum*, a Ulleung Island endemic, had an estimated divergence time from its continental lineage at 0.29 Ma (95% HPD, 0.08–0.59 Ma). Lastly, the split of *A. macrostemon* from its sister lineage (*A. schoenoprasoides* and *A. caeruleum* in sect. *Pallasia* and *Caerulea*, respectively) was estimated at 4.55 Ma (95% HPD, 1.87–7.54 Ma), whereas *A. condensatum* and *A. chinense* shared their most recent common ancestor at 7.62 Ma (95% HPD, 3.02–12.36 Ma).

## 4 Discussion

### 4.1 *Allium* plastome variation and evolution

In this study, we newly sequenced a total of 18 species (20 accessions) of *Allium* plastomes, primarily focusing on two subgenera, *Rhizirideum* and *Anguinum*, and found them highly conserved in genome structure and organization. The genome size of the newly sequenced 20 plastomes ranged from 153,121 (*A. ochotense*; Japan) to 154,049 bp (*A. ulleungense*), which was within the broad range of the genus (145 to 160 kb; Xie et al., 2020 and references therein) (**Table 1**). The GC contents of the 20 plastomes varied from 36.8% to 37.1%, which was within the range of the genus, that is, from 36.7% to 37.8% (Xie et al., 2020). In addition, the gene number (protein coding, tRNAs, and rRNAs) was very similar to those reported in previous *Allium* plastomes, suggesting overall high conservation of plastomes as previously shown within infrageneric and infrafamilial levels in angiosperms (e.g., Aroideae, Henriquez et al., 2020; *Hosta*, Yang et al., 2021; Crassulaceae, Chang et al., 2021; *Cotoneaster*, Yang et al., 2022; *Ocotea*, Trofimov et al., 2022).

We examined the position of SC/IR borders across the three evolutionary lines in the 84 *Allium* plastomes (**Supplementary Figure 1**). As shown previously in angiosperm plastomes, gene contents on both sides of the SC/IR borders of *Allium* plastomes were conserved (Downie and Jansen, 2015). Mostly, *rpl22* was interrupted by the LSC/IRb border in *Allium*, but its position entirely within the LSC region has occurred multiple times in different lineages/species of three evolutionary lines (**Supplementary Figure 1**). In addition, the interrupted position of *rps19* in the IRa/LSC border has occurred stochastically in different species within *Allium*: *A. kingdonii* (subg. *Cyathophora*), *A. fetisowii* (subg. *Melanocrommyum*), *A. victorialis* (subg. *Anguinum*), *A. mairei*/*A. spicatum* (subg. *Cyathophora*), and *A. tenuissimum* (subg. *Rhizirideum*). For SSC/IR boundaries, two SSC/IR borders crossed two *ycf* genes in most of the *Allium* plastomes and a large part of the *ycf1* sequence was mainly positioned in the IRb region. In addition to the *ndhF* gene’s entire location within the SSC region, its location at the boundary of SSC and IRb was also occurred stochastically. It remains to be determined whether plastome structure reflects phylogenetic or stochastic events, based on broader taxon sampling, ecological analysis, and nuclear-based phylogenomics (Scobeyeva et al., 2021).

We also found five highly variable regions across the three evolutionary lines of *Allium* (**Figure 3**). The mutation hotspots included two genic regions, *ycf1* (Pi = 0.0448) and *ndhF* (Pi = 0.03192). We further identified the three intergenic regions (*trnS*/*trnG*, *rps15*/*ycf1*, and *rbcL*/*accD*). Of eight mutation hot spots found among 18 species of *Allium* (Namgung et al., 2021), the same three regions (i.e., *ycf1*, *ndhF*, and *rps15*/*ycf1*) were also found in the current study. Two additional intergenic regions, *rps15*/*ycf1* and *rbcL*/*accD*, showed high variability. The same three loci (*ycf1*, *trnS*/*trnQ*, and *rbcL*/*accD*) were found to be highly variable chloroplast markers for evaluating plant phylogeny at low taxonomic levels and for DNA barcoding in angiosperms (Dong et al., 2012). Three regions, *ycf1*, *trnS*/*trnG*, and *rps15*/*ycf1*, have been determined to be highly variable among the 48 *Lilium* (Liliaceae) plastomes (Sheikh-Assadi et al., 2022). However, different sets of highly variable chloroplast regions (with exception in one or two loci depending on taxa) were identified among the eight species of *Fritillaria* (Liliaceae; Bi et al., 2018) and the six species of *Hosta* (Amaryllidaceae; Yang et al., 2021) in monocots, cautiously limiting the wide applicability of universally hypervariable chloroplast regions. Nonetheless, highly variable chloroplast regions found in this study can be used to resolve phylogenies within *Allium* and for DNA barcoding or phylogeographic study of closely related species or related genera in monocots.

### 4.2 Inter-subgeneric relationships within *Allium*

Owing to the insufficient sectional-level sampling in this study, our discussion is based primarily on phylogenetic relationships among subgenera reported in previous studies (Friesen et al., 2006; Li et al., 2010). Our study independently confirmed the three evolutionary lines and their relationships in the genus *Allium* (Friesen et al., 2006; Li et al., 2010; Xie et al., 2020) (**Figure 4A, B**). In particular, the previous plastome-based phylogeny (Xie et al., 2020) was robustly recovered in this study. The first evolutionary line includes three subgenera, *Nectaroscordum*, *Microscordum*, and *Amerallium* (**Figure 4A**). For inter-subgeneric relationships within this lineage, subg. *Nectaroscordum* diverged first and then was followed by two sister subgenera, *Microscordum* and *Amerallium*, corroborating with a previous study (Friesen et al., 2006). This tree topology further supports the view of descending aneuploidy basic chromosome number, *Nectaroscordum* (x = 9), *Microscordum* (x = 8), and *Amerallium* (x = 7): ascending basic chromosome number also evolved independently in several morphologically derived *Amerallium* groups (Friesen et al., 2006). Although the second evolutionary line was also strongly recovered, inter-subgeneric relationships appeared to be different between the current and previous studies (**Figure 4A**). For example, despite the lack of representatives from two subgenera (*Porphyroprason* and *Vvedenskya*), the complete plastome tree strongly suggests that *Caloscordum* is closely related to *Melanocrommyum* and that *Anguinum* represents another distinct lineage within this evolutionary line. The previous study based on ITS sequences suggests that *Caloscordum* represents the first diverged subgenus followed by *Anguinum*, the two sister subgenera *Porphyroprason* and *Vvedenskya*, and the massive radiation of *Malanocrommyum* (Friesen et al., 2006). Subgenus *Caloscordum* is an oligotypic group with three species in East Asia and shares several characteristics with subg. *Melanocrommyuum* (that is, multiovulate locules, subterraneous leaf sheaths, and the presence of relatively large inner vascular bundles in the scapes; Friesen et al., 1986; Fritsch, 1993), further supporting our current plastome relationship. In addition, similar characteristics of seed testa cells, slightly verrucose periclinal wall, and straight anticlinal walls shared between *Anguinum* and *Caloscordum* might be viewed as either convergent evolution or symplesiomorphies (Friesen et al., 2006). It is required to include the two monotypic subgenera *Porphyroprason* (*A. oreophilum*) and *Vvedenskya* (*A. kujukense*), which have several autapomorphies, to fully determine the inter-subgeneric relationships within this evolutionary lineage.

The third evolutionary line is rather complex compared with the two former ones (**Figure 4B**). One consistent relationship found in the current and previous studies is an early divergence of two subgenera, *Butomissa* and *Cyathophora*, within this evolutionary line. Subgenus *Butomissa*, a small group comprising two subgroups, occurs in the Siberian-Mongolian-North Chinese steppes and the mountains from eastern to central Asia up to the borderline of the eastern Mediterranean area (Friesen et al., 2006). The second diverged lineage, subg. *Cyathophora*, is a small and solely Asian (Tibet and the Himalayas) group, and it shares certain characteristics (i.e., one row of identically oriented vascular bundles in the leaf blades, the presence of palisade parenchyma and subcortical laticifers, and biovulate locules) (Fritsch, 1988; Hanelt, 1992; Friesen et al., 2006). The remaining clade of the five subgenera *Cepa*, *Reticulatobulbosa*, *Polyprason*, *Rhizirideum*, and *Allium* (100% BS), is further complicated by non-monophyly of all but one subgenus *Rhizirideum* (**Figure 4B**). Nevertheless, after the divergence of one clade of subg. *Allium*, the following major lineages were identified: (1) one lineage of subg. *Cepa* and subg. *Reticulatobulbosa*, (2) subg. *Rhizirideum*, and (3) one lineage of subg. *Reticulatobulbosa*, one lineage of subg. *Allium*, one lineage of subg. *Cepa*, and two lineages of subg. *Polyprason*. With much broader sampling, it is necessary to determine phylogenetic relationships among five subgenera to reflect their relationships in the classification system and reevaluate key characteristics in the context of the new phylogenetic framework.

We estimated the crown age of the genus *Allium* at 23.65 Ma (95% HPD, 11.97–35.31 Ma), which is similar to an earlier estimate at 22.18 Ma (95% HPD, 15.23–34.81 Ma) (Xie et al., 2020) (**Figure 5**). The crown ages of the three evolutionary lines were estimated to be 20.13 Ma (first line), 13.88 Ma (second line), and 17.05 Ma (third line), which were roughly the same time scale as in previous studies (Xie et al., 2020). These suggest that common ancestors of three evolutionary lines existed during the middle to early Miocene. We further provided some other important divergent times estimated in this study, and they included (1) the split of subg. *Microscordum* from subg. *Amerallium* at 17.98 Ma (95% HPD, 7.38–27.81 Ma), (2) crown age of 13. 75 Ma (95% HPD, 5.89–21.96 Ma) for subg. *Amerallium*, (3) crown age of 13.08 Ma (95% HPD, 5.81–21.50 Ma) for the clade of subgenera *Caloscordum* and *Melanocrommyum*, (4) crown age of 10.72 Ma (95% HPD, 3.99–18.70 Ma) for subg. *Butomissa*, (5) crown age of 7.47 Ma (95% HPD, 2.87–13.34 Ma) for subg. *Cyathophora*, and (6) crown age of 5.49 Ma (95% HPD, 2.47–8.34 Ma) for the major lineage of subg. *Polyprason* (sect. *Daghestanica*). These estimates suggest that major subgenera divergence or speciation events occurred during the Miocene, providing useful information for future studies on subgenera and sections.

### 4.3 Species relationships within subg. *Anguinum*

In this study, we confirmed the two major lineages within subg. *Anguinum* (Herden et al., 2016) (**Figure 4A**). The novel finding, based on the plastome phylogeny, was the phylogenetic position of *A. tricoccum*, which exclusively occurs in eastern North America. Unlike previous studies, two accessions of *A. tricoccum* formed monophyly (100% BS) and clearly belonged to the Eurasian lineage (Group A; 100% BS). These results support the recognition of the Eurasian-American lineage and East Asian lineage but refute the phylogenetic incongruence between nuclear ITS and chloroplast. The previous chloroplast phylogeny based on three non-coding regions (*rpl32*-*trnL* spacer, *rps16* intron, and *atpB*-*rbcL* spacer) showed that *A. tricoccum* is sister to *A. prattii* from China, which belongs to the East Asian lineage (group B). The cloning of nrDNA ITS sequences found three ribotypes, that is, *A. tricoccum* ribotype, the East Asian (Group B) ribotype, and the Eurasian-American (group A) ribotype, and subsequently suggested a hybridization event between the A and B groups (Herden et al., 2016). The frequencies of three ribotypes showed that all but one clone belonged to group A: the first type (two clones) belongs to *A. tricoccum* (group A), the second type (one clone) belongs to group B, and the third type (13 clones) is related to group A. Therefore, according to the current robust placement of *A. tricoccum* in group A, incomplete homogenization of different ribotypes based on concerted evolution (i.e., deep coalescence, incomplete lineage sorting) is a more likely explanation than reticulation event between the two groups (Feliner and Rossellö, 2012). Consequently, the congruent tree topology between nuclear and chloroplast phylogeny suggested that the intercontinental disjunction must have occurred soon after the initial divergence between the two major groups of subg. *Anguinum* and further confirmed the Eurasian-American lineage (Herden et al., 2016). After the initial disjunction event in group A, the lineage of *A. ulleungense* on Ulleung Island and Chinese *A. listera* diverged from the remaining lineage, which subsequently diversified to three extant species (*A. victorialis*, *A. ochotense*, and *A. microdictyon*).

There has been considerable confusion on the taxonomy in the subg. *Anguinum* due to geographical distribution patterns of three species: *A. victorialis*, *A. microdictyon*, and *A. ochotense* (Herden et al., 2016). Nevertheless, those three species are unanimously accepted based on the current species concept and distribution areas given by Prokhanov (1930), with a recent new species recognition of *A. ulleungense* on Ulleung Island from more widely distributed *A. ochotense* (Choi et al., 2019). Previous phylogenetic studies provided very few resolutions to fully understand species boundaries and phylogenetic relationships. For example, the ITS tree showed four major clades (*A. ulleungense*, *A. tricoccum*, *A. victorialis*, and *A. ochotense*–*A. microdictyon*–*A. listera*), whereas the combined cpDNA showed *A. ulleungense*, *A. microdictyon*, and *A. victorialis*–*A. ochotense* (Herden et al., 2016; Choi et al., 2019). The complete plastome sequences in this study suggest the possibility of non-monophyly of two species, *A. ochotense* and *A. microdictyon* and that *A. microdictyon* shares its most common ancestor with *A. ochotense*, which is geographically much closer than *A. victorialis* (strictly European) (**Figure 4A**). The close relationship between *A. ochotense* and *A. microdictyon* is further corroborated by the ITS sequences: two species are part of a highly unresolved clade, such as *A. listera* (88% BS and 0.98 PP), and *A. victorialis* is sister to this highly unresolved clade (98% BS and 0.99 PP) (Herden et al., 2016).

One more important finding of this study is that *A. ulleungense*, endemic to Ulleung Island, Korea, is a taxonomically distinct entity (**Figure 4A**). It was previously recognized as either *A. victorialis* (Yu et al., 1981) or *A. ochotense* (Choi and Oh, 2011). Although it also resembles *A. microdictyon* morphologically, *A. ulleungense* has much broader leaves, larger whitish perianth, and is diploid (2n = 2x = 16) (Choi et al., 2019). The current study, which is based on the complete plastomes, suggests a sister relationship between *A. ulleungense* and *A. listera*, which are both diploid (2n = 2x = 16) (Jing et al., 1999). This clade is in turn sister to the clade containing Eurasian species (*A. victorialis*, *A. microdictyon*, and *A. ochotense*), and because of the phylogenetic incongruence between previous ITS trees (Herden et al., 2016; Choi et al., 2019) and our current plastome tree, further study is required to gain insights into ITS ribotype evolution and determine the precise phylogenetic position of *A. ulleungense*. Based on the complete plastome sequences in this study, slightly younger crown age of subg. *Anguinum* at 4.85 Ma (95% HPD, 2.08–8.32 Ma) was estimated compared with that based on ITS sequences (5.44 Ma, 95% HPD, 2.13–9.4 Ma; Herden et al., 2016). The intercontinental disjunct event and the split of Ulleung Island endemic *A. ulleungense* from its continental sister species were estimated to be 2.71 Ma (95% HPD, 1.00–4.86 Ma) and 1.57 Ma (95% HPD, 0.05–4.43 Ma), which occurred in late Pliocene (Piacenzian) and mid Pleistocene (Calabrian), respectively. Further study is required to (1) test the monophyly of each species and their phylogenetic relationships, (2) assess the polyploidization process in *A. ochotense* (2n = 4x = 32), and (3) understand geographical disjunct distribution patterns of *A. microdictyon* in Caucasus, Siberia (from West-to-Central and East Siberia), Southern Ural area, North Mongolia, Kazakhstan, and Korea.

### 4.4 Evolution within subg. *Rhizirideum*

The inter-sectional and species relationships within subg. *Rhizirideum* were not well resolved in previous studies (Nguyen et al., 2008; Li et al., 2010; Sinitsyna et al., 2016; Jang et al., 2021). Although the position of sect. *Eduardia* in the third evolutionary line of *Allium* is yet to be determined, it appears that two sections, *Tenuissima* and *Rhizirideum*, are monophyletic (Li et al., 2010). The current study, which is based on the complete plastome sequences, showed that sect. *Caespitosoprason* appears not to be monophyletic, but two major groups within the monophyletic sect. *Rhizirideum*, “Asiatic” and “European” groups (Sinitsyna et al., 2016), are strongly recognized (100% BS each), except for *A. nutans* (**Figure 4B**). It was shown that within the Asiatic group, three subgroups were inferred based on two chloroplast region sequences (*trnQ*-*rps16* and *trnL*-*rpl32*; Sinitsyna et al., 2016): (1) unresolved *A. spirale* (2x)–*A. spurium* (4x), (2) clade of *A. nutans* (4x)–*A. senescens* (4x and 6x)–*A. azutavicum* (unknown ploidy) (0.88 PP and 68% BS), and (3) *A. austrosibiricum* (2x)–*A. rubens* (4x)–*A. tytthocephalum* (4x)–*A. tuvinicum* (2x)–*A. prostratum* (2x)–*A. minus* (2x)–*A. burjaticum* (4x)–*A. stellerianum* (4x) (0.99 PP and 78% BS). ITS phylogeny, which is based on diploid species only, showed that very little was resolved within the Asiatic group, other than three species, *A. austrosibiricum*, *A. prostratum*, and *A. minus*, which shared their most recent common ancestor (0.97 PP and 80% BS). Several incongruences between ITS and chloroplast phylogeny were as follows: (1) *A. nutans* (4x) is closely related to *A. senescens* (4x, 6x)/*A. azutavicum* in the cpDNA tree (0.86 PP and 62% BS), but it is sister to *A. austrosibiricum* (2x) in the ITS tree (0.89 PP and 89% BS); (2) *A. tyttocephalum* (4x) is closely related to *A. rubens* (2x) in the cpDNA tree (0.86 PP and 62% BS), but it is unresolved in the ITS tree; and (3) *A. minus* is closely related to some accessions of *A. prostratum* (2x) (0.88 PP and 58% BS), but it is unresolved in the ITS tree (Sinitsyna et al., 2016). The narrow Korean endemic *A. minus* is sister to *A. senescens* (Mongolian accession) in this study, suggesting its potential origin from wild onion native to Mongolia. *Allium minus*, which occurs in the type locality only (Taegisan Mountain, Walhaksan Mountain, Gangwon-do Province, and Yangju, Gyeonggi-do Province) in Korea, was initially recognized as a variety of *A. senescens* (Yu et al., 1981). Based on the detailed phylogeographic study, it remains to be determined as to how diploid *A. minus* in Korea is related to tetraploid and hexaploid *A. senescens* outside of the Korean Peninsula. Recently, populations of *A. senescens* on Ulleung Island were described as a new tetraploid endemic species (*A. dumebuchum*) in Korea (Jang et al., 2021). Owing to its distinct diagnostic features (bigger floral parts and late flowering time) from its closely related species (i.e., *A. spirale*, *A. spurium*, *A. minus*, and *A. senescens*), *A. dumebuchum* was described as a new taxon; however, its phylogenetic position was unclear (Jang et al., 2021). The current plastome tree suggests strongly that *A. dumebucum* is sister to the clade containing *A. austrosibiricum*, *A. senescens*, and *A. minus*, suggesting that the former diverged earlier than the three latter ones. The split of *A. dumebucum* from the continental lineage was estimated to be 0.29 Ma (95% HPD, 0.08–0.59 Ma) in Pleistocene (Ionian), much younger than the other Ulleung Island endemic *A. ulleungense* (1.54 Ma). The crown age of subg. *Rhizirideum* was estimated to be 8.41 Ma (95% HPD, 3.85–13.49 Ma) in the late Miocene, much older than that of subg. *Anguinum*. This estimate is comparable with that based on ITS sequences (7.15 Ma; Sinitsyna et al., 2016). However, the split between “Asian” and “European” groups in this study was estimated to be much younger at 2.18 Ma (95% HPD, 0.75–4.13 Ma) compared with that based on ITS sequences (ca. 3.97 Ma, 95% HPD, 1.13–6.91 Ma; Sinitsyna et al., 2016). Moreover, during the Pleistocene, interspecific hybridization and polyploidization may have led to the diversification of *Allium* species in sect. *Rhizirideum*.

### 4.5 Phylogenetic position of sect. *Condensatum*

As one of the morphologically “difficult” species in variable sections of *Scorodon*, *Reticulatobulbosa*, and *Oreiprason* (Friesen et al., 2006), the phylogenetic position of *A. condensatum* (**Figure 1E**), which occurs from eastern Siberia and Mongolia to North Korea and Russian Far East, has been elusive. Based on one of the four major clades in sect. *Oreiprason*, the east Asian monotypic section *Condensatum* was newly recognized along other sections (*Cepa*, *Schoenoprason* sensu stricto, *Annuloprason*, and *Sacculiferum*) in polyphyletic subg. *Cepa* (Friesen et al., 2006; Li et al., 2010). However, previous phylogenetic studies have shown the uncertain position of *A. condensatum*. For example, the nuclear ITS sequence-only tree showed that *A. condensatum* is sister to the clade containing several sections of subg. *Allium* (0.52 PP and <50% BS; Li et al., 2010) or highly unresolved compared with other subgenera (Friesen et al., 2006; Nguyen et al., 2008). In addition, the combined nuclear ITS and chloroplast *rps16* sequences, which focused on Chinese *Allium*, placed *A. condensatum* in the highly unresolved clade of the third evolutionary lineage, such as subgenera *Cepa*, *Polyprason*, *Allium*, and *Reticulatobulbosa* (Li et al., 2010). Alternatively, the combined and nuclear ITS and chloroplast *trnL–trnF*, which focused on Korean/northeastern Chinese/Canadian representative species, placed *A. condensatum* sister to the clade of subg. *Allium* (<50% BS; Choi et al., 2012). All the previous studies highlighted the uncertain phylogenetic position of *A. condensatum*, and for the first time, based on the complete plastome sequences, we demonstrated that *A. condensatum* is closely related to *A. chinense*, which is widely cultivated as a vegetable in tropical and subtropical China (100% BS). It was also estimated that the two species shared their most recent common ancestor at 7.62 Ma (95% HPD 3.02–12.36 Ma) in the late Miocene. Although the sister relationship is strongly supported based on the current study, the two species have numerous differences, such as perianth color (pale purple to dull purple for *A. chinense* vs. pale yellow for *A. condensatum*) and scape (lateral, 20–40 cm in length for *A. chinense* vs. central, 30–80 cm for *A. condensatum*), and they are closely related to the species of subg. *Reticulatobulbosa* (91% BS) (**Figure 4B**). It was not possible to verify the voucher of *A. chinense* (NC043922; Yang et al., 2019), and this close relationship could be due to the potential sampling limitation of the current study. Therefore, this preliminary placement of *A. condensatum* close to subg. *Cepa* and sect. *Sacculiferum* rather than *Allium* species from sect. *Cepa*. is still tentative and remains to be determined based on extensive sampling of *Allium* plastomes.

### 4.6 Phylogenetic position of *A. macrostemon*

*Allium macrostemon* (**Figures 1Q–T**) occurs commonly in sunny lowland meadows, forest margins, and mountain foothills of Russia (Far East), Mongolia, China, Taiwan, Korea, and Japan. The current study showed that *A. macrostemon* (at least based on accessions from China and Korea) is monophyletic and shared its most recent common ancestor with *A. caeruleum* (subg. *Allium* sect. *Caerulea*) and *A. schoenoprasoides* (subg. *Allium* sect. *Pallasia*). This *Allium* clade is distinct from the second *Allium* clade, which includes *A. ferganicum*, *A. ampeloprasum*, and *A. sativum* (**Figure 4B**). *Allium macrostemon* was treated as a member of subg. *Allium* sect. *Allium* (Li et al., 2010), but potential recognition under a new section was suggested (Friesen et al., 2006). *Allium macrostemon* shares similar testa sculptures and morphological characteristics (that is, bulbil development in inflorescence, pistil morphology, and seed shape) with members of sect. *Caerulea*, especially with *A. caeruleum* (Choi et al., 2012). As two sister lineages were recognized at the sectional level (*Caerulea* and *Pallasia*), it is reasonable to propose a new section for *A. macrostemon*, which is restricted to East Asia. Furthermore, molecular dating suggests that the divergence of *A. macrostemon* from the central Asian lineage occurred earlier (4.55 Ma, 95% HPD 1.87–7.54 Ma) than the split between the two sections of *Caerulea* and *Pallasia* from Central Asia (**Figure 5**). Thus, in this study, we newly described a monotypic section with a type species of *A. macrostemon* that occurs in East Asia.

*Allium* subgen. *Allium* sect. ***Macrostemon*** S.-C.Kim & H.J.Choi, sect. nov.-TYPE: *A. macrostemon* Bunge

**Description.** Bulbs solitary with short rhizome, subglobose, sometimes with bulbels; tunics membranous. Leaves fragile, linear, minutely angular, adaxially channeled, hollow in cross section. Umbel globose, sometimes replaced totally or partially by pseudoviviparous bulbils. Perianth widely spreading, whitish pink; inner tepals slightly longer than outer ones; filaments slightly exserted, subulate; anthers elliptical; ovary subcubical, green, with hood-like projections at base; ovules two per locule; style exerted; stigma smooth. Capsules trigonous; seeds oval, flat in cross section.

## Data availability statement

The datasets presented in this study can be found in online repositories. The names of the repository/repositories and accession number(s) can be found below: https://www.ncbi.nlm.nih.gov/genbank/, OP743930-OP743948 https://www.ncbi.nlm.nih.gov/genbank/, OP754898.

## Author contributions

H-JC and S-CK conceived and designed the study; JY and S-HK conducted the experiment; H-JC, H-YG, and S-CK provided experimental support; JY and S-HK prepared the original draft; and H-YG, H-JC, and S-CK revised and finalized the manuscript. All authors contributed to the article and approved the submitted version.
