# Supplementary material for: New insights into the phylogenetic relationships among wild onions (Allium, Amaryllidaceae), with special emphasis on the subgenera Anguinum and Rhizirideum, as revealed by plastomes
Source: Front Plant Sci. 2023 Mar 21;14:1124277. doi: 10.3389/fpls.2023.1124277 (PMC10070991; doi:10.3389/fpls.2023.1124277)

Supplementary Material

New insights into the phylogenetic relationships among wild onions (*Allium*, Amaryllidaceae), with special emphasis on the subgenera *Anguinum* and *Rhizirideum*, as revealed by plastomes

**JiYoung Yang^1^, Seon-Hee Kim^2^, Hee-Young Gil^3^, Hyeok-Jae Choi^4^*, Seung-Chul Kim^5^***

*** Correspondence:**Hyeok-Jae Choi and Seung-Chul Kim

hjchoi1975@changwon.ac.kr; sonchus96@skku.edu

**Supplementary Figure 1**. Comparison of the border positions of the large single copy (LSC), small single copy (SSC), and inverted repeat (IR) regions among the 84 *Allium* plastomes. Gene names are indicated in boxes, and their lengths in the corresponding regions are displayed above the boxes. ***Ψ*** indicates a pseudogene. Deletion of *rps19* gene is shown dashed line.


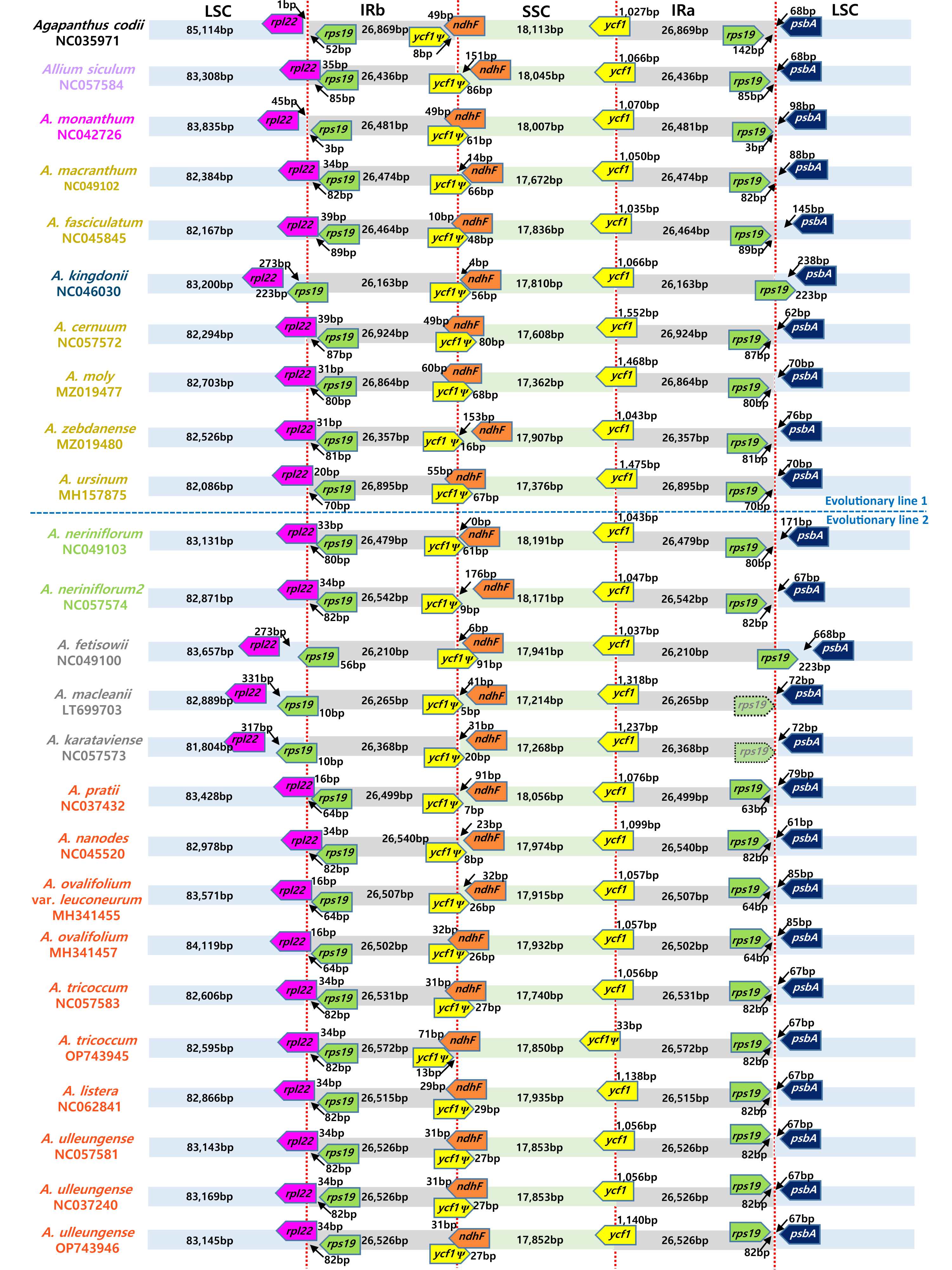


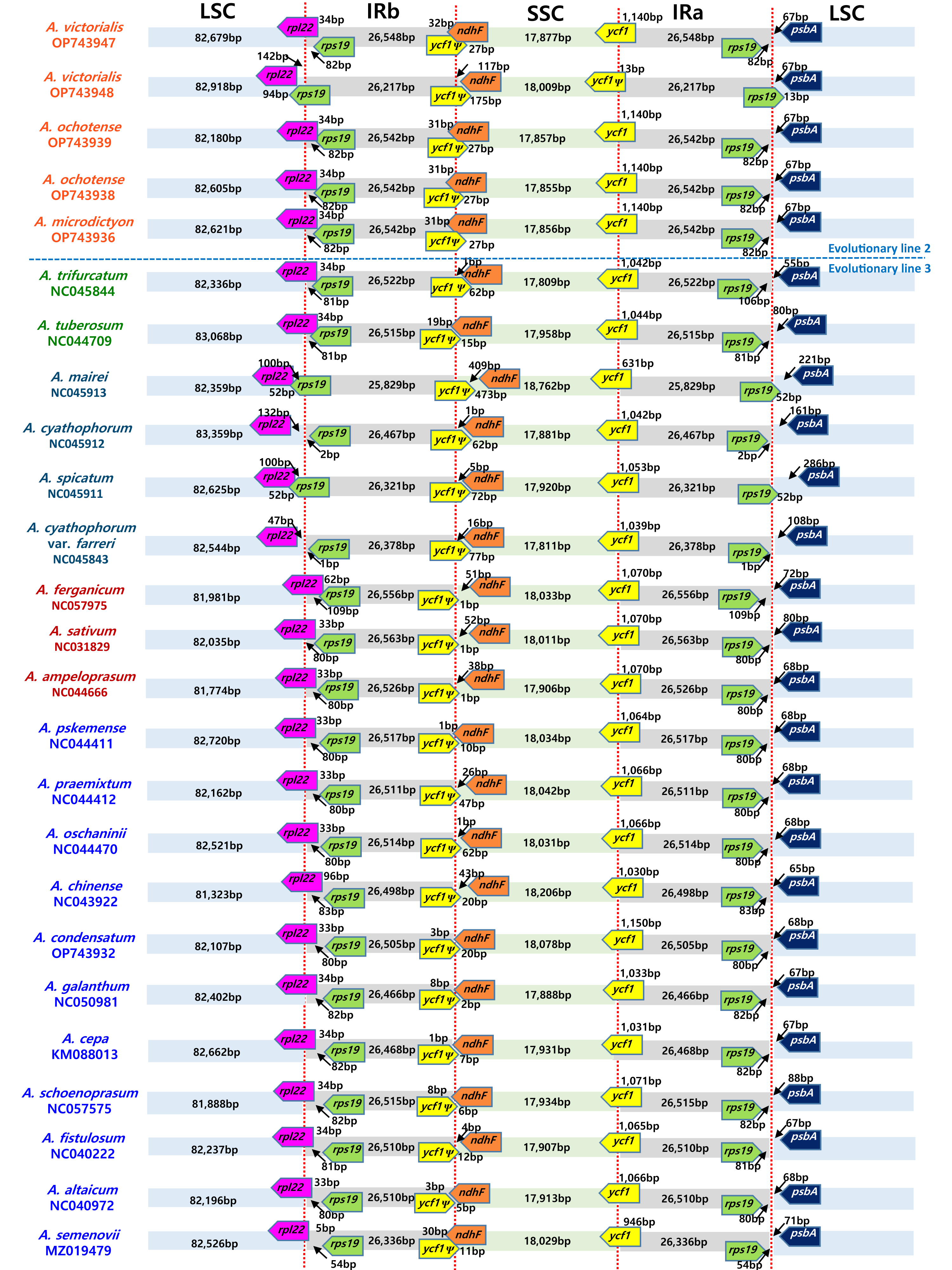


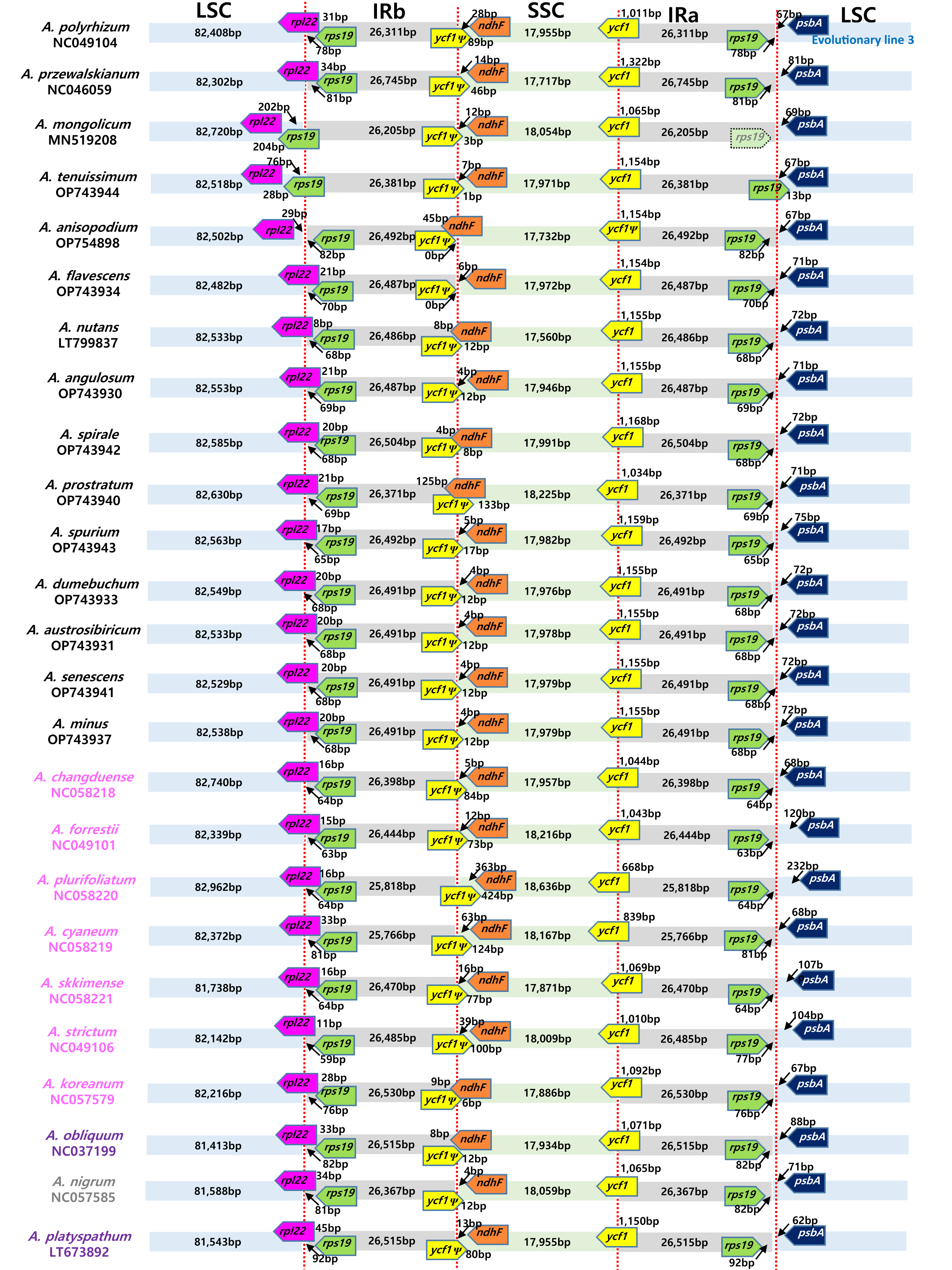


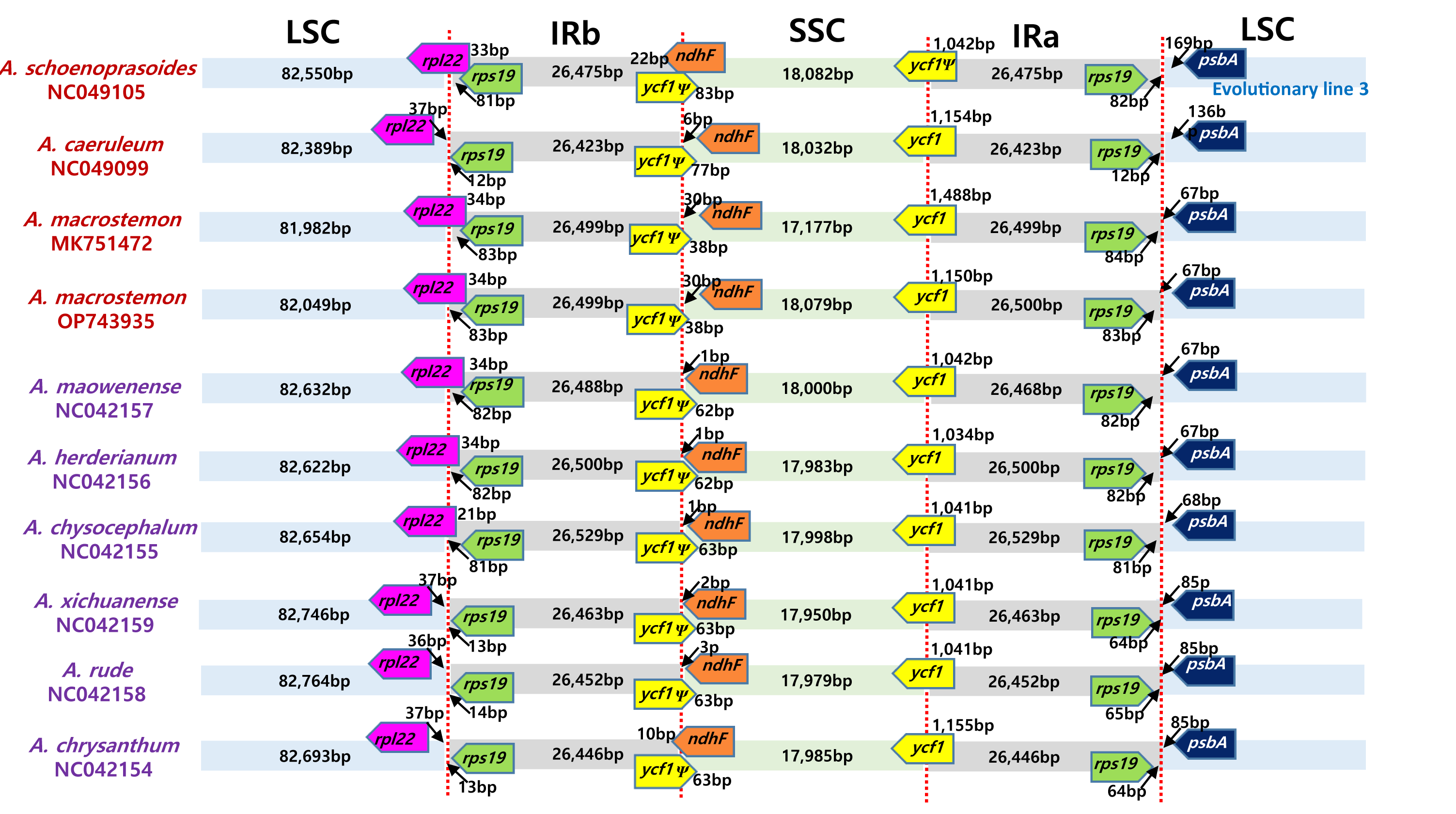

Supplement: Supplementary file 1 [file Presentation_1.zip › Supplementary Figure 1.docx]
